# Activity of PD1 inhibitor therapy in advanced sarcoma: a single-center retrospective analysis

**DOI:** 10.1186/s12885-020-07021-x

**Published:** 2020-06-05

**Authors:** Dionisia Quiroga, David A. Liebner, Jennifer S. Philippon, Sarah Hoffman, Yubo Tan, James L. Chen, Scott Lenobel, Paul E. Wakely, Raphael Pollock, Gabriel Tinoco

**Affiliations:** 1 The Ohio State University Comprehensive Cancer Center, The Ohio State University, 410 W 12th Avenue, Columbus, OH 43210 USA; 2 Department of Internal Medicine, Division of Medical Oncology, The Ohio State University, Starling Loving Hall, 320 W 10th Ave, Columbus, OH 43210 USA; 3 Department of Biomedical Informatics, The Ohio State University, 250 Lincoln Tower, 1800 Cannon Dr, Columbus, OH 43210 USA; 4 Center for Biostatistics, The Ohio State University, 2012 Kenny Rd, Columbus, OH 43221 USA; 5 Department of Radiology, The Ohio State University, 410 W 12th Avenue, Columbus, OH 43210 USA; 6 Department of Pathology, The Ohio State University, 410 W 10th Ave, Columbus, OH 43210 USA; 7 Department of Surgery, The Ohio State University, 410 W 10th Ave, Columbus, OH 43210 USA; 8 The Ohio State University Comprehensive Cancer Center, 320 W 10th Ave, A444 Starling Loving Hall, Columbus, OH 43210 USA

**Keywords:** Immunotherapy, Soft tissue sarcomas, Retrospective analysis

## Abstract

**Background:**

Sarcomas constitute a heterogeneous group of tumors with different clinical behaviors and variable responses to systemic therapies. Recent immunotherapy studies with PD1 inhibitors (PD1i) show promising results with use in certain soft-tissue sarcomas; however, the clinical and molecular features that best predict response to PD1i remain unclear.

**Methods:**

Demographic, imaging, histologic, and genetic sequencing data was collected for sarcoma patients who received nivolumab or pembrolizumab (PD1i) treatment at our institution between January 1st 2015 and April 23rd 2018. The primary objective was to determine progression-free survival (PFS) in patients with advanced sarcomas receiving PD1i. Secondary objectives included determining overall survival (OS) and assessment of characteristics associated with response to PD1i. Fifty-six patients who were treated with PD1i therapy met inclusion criteria for this study.

**Results:**

Partial response towards PD1i treatment was seen in 3 in 26 evaluable patients, but no complete responses were observed (overall response rate 11.5%). Within this group of patients, the 90 day PFS was found to be 48.8%. In patients in whom PD1 expression was known, there was a statistically significant positive correlation between expression of PD1 and longer PFS and OS rates. Patients that were treated with more than four cycles of PD1i therapy were also more likely to have a greater OS.

**Conclusions:**

This study suggests activity of PD1i in a pretreated cohort of advanced sarcoma patients, particularly for the subset of patients with PD1 positive tumors. Our results highlight the importance of further research to better target the optimal patient population and markers of response.

## Background

Sarcomas represent a diverse group of soft-tissue and bone neoplasms of mesenchymal origin, with different morphologic and genetic features as well as variable clinical behaviors for which there are currently a limited number of therapeutic options [1]. There are approximately 16,000 new sarcoma cases diagnosed in the United States every year, with an estimated 5000 related deaths [2]. About one-third of sarcomas are diagnosed in those under the age of 45, while only one-tenth of all cancers occur in this age group [2]. Therefore, although sarcomas are rare, their societal impact from person-years lost due to related deaths and from long-term treatment effects is considerable. While locally resectable sarcomas can be cured surgically (or using a multimodality approach with perioperative chemotherapy and radiation therapy), a large proportion of sarcomas are already at advanced stages upon diagnosis [3]. For the majority of advanced sarcomas, the overall prognosis is dismal and enrollment in clinical trials is encouraged [2]. Chemotherapy with single agents, anthracycline-based combinations, or other agent combinations have been widely used for patients with advanced, unresectable, and metastatic disease, albeit with limited benefit [4–27]. The efficacy of these treatments is even further restricted when used as second-line or later systemic therapies [28]. Thus, there is an urgent need to explore new therapeutic options that could improve outcomes with fewer side effects.

Checkpoint inhibitors (anti-PD1, anti-PDL1, and anti-CTLA-4 antibodies) have become an appealing new option for the treatment of several advanced cancers, and are now first-line and/or second-line therapies for non-small cell lung carcinoma, melanoma, and renal cell carcinomas [29–31]. A strong association between PD1/PDL1 expression and response to PD1 and PDL1 inhibitors has previously been established in several tumor types; however, the role of checkpoint inhibitors in sarcoma treatment is unclear. Interestingly, the analysis of various sarcoma tissue samples have shown a significant positive correlation between sarcomas that express PD1/PDL1 and those that have increased T cell infiltration and activation [32, 33]. Moreover, patients whose sarcomas contain increased copy numbers of the PD1 gene have poorer survival outcomes [34]. The optimal marker of response to immunotherapy in sarcoma patients remains uncertain.

So far, there have been two landmark studies of immunotherapy use in sarcoma. First, SARC0238, a phase II, single-arm study was conducted on soft-tissue and bone sarcoma patients who received pembrolizumab treatment every 3 weeks and monitored for disease progression and overall mortality [35]. This study showed promising tumor regression in several patients, particularly those with undifferentiated pleomorphic sarcomas (UPS) or dedifferentiated liposarcomas (LPS). Next, the Alliance A091401 trial was designed to study the role of dual checkpoint inhibitors in patients with metastatic sarcoma [36]. The results of this study revealed that nivolumab combined with ipilimumab demonstrated promising efficacy in certain sarcoma subtypes.

Further studies are essential to assess the response of sarcomas to checkpoint inhibitors as well as determine patient factors that are associated with checkpoint inhibitor response. We performed a retrospective analysis of 56 sarcoma patients treated at our institution with PD1 inhibitors (PD1i), determined progression-free survival (PFS) and overall survival (OS), and correlated patient demographic factors with their survival rates.

## Methods

### Patients and study design

This was a single-institution, retrospective cohort study of adult patients with any stage sarcoma treated at The Ohio State University Medical Oncology Sarcoma Clinic between the dates of January 1st, 2015 and April 23rd, 2018 who received at least one dose of PD1i (pembrolizumab or nivolumab). Patients who were receiving both PD1i with a secondary cancer treatment (e.g. tyrosine kinase inhibitor) were allowed. Exclusion criteria included current clinical trial enrollment at the time of their treatment with checkpoint inhibitor treatment. Treatment history, demographic, genetic, pathologic, and radiologic information of these patients were retrospectively collected through a review of their electronic medical records. Intratumoral PD1 and PDL1 expression were considered positive if ≥1% of cells collected stained positive for these markers. Ethical approval of this study was obtained from The Ohio State University Comprehensive Cancer Center institutional review board (IRB protocol No. 2017C0063).

### Statistical analysis

Tumor measurements of lesions identified on patient CT or MRI imaging were made by a board-certified radiologist (SL), and clinical responses to PD1i treatment were assessed according to Response Evaluation Criteria in Solid Tumors version 1.1 (RECIST 1.1). In order for a patient to be included in PFS analyses, it was required that they have a baseline scan within 6 weeks of initiating PD1i. Additionally, a follow-up scan at least 4 weeks after initiation of PD1i therapy was required, and imaging progression must have been deemed eligible for analysis as per RECIST 1.1. Best tumor response was evaluated by RECIST 1.1 criteria and defined as the most significant decrease in target tumor burden. Kaplan-Meier analyses were used to compare PFS and OS rates. For analysis of PFS in patients who were lost to follow-up or who were alive without PFS at the end of this study, data was censored at the time of their last tumor imaging. OS was calculated from the day that their PD1i was started until their reported death date. For analysis of OS, patients who were known to have been alive at the end of the study period were censored at this endpoint (April 1st, 2020). Patient groups were compared by log-rank (Mantel-Cox) analysis. All statistical analyses were completed through use of GraphPad software (GraphPad Software, La Jolla, CA, USA).

## Results

### Patient demographics

A total of 56 patients were included in this study. Among these participants, 58.9% were men, the median age was 55.5 years old, and the vast majority identified as Caucasian (91.1%) (Table 1).

**Table 1:** Patient demographics

	N (%)
**Age at time of diagnosis (years)**	
Younger than 50	21 (37.5%)
50 or older	35 (62.5%)
**Sex**	
Male	33 (58.9%)
Female	23 (41.1%)
**Race/Ethnicity**	
White/Caucasian	51 (91.1%)
African American	2 (3.6%)
Latino/Hispanic	2 (3.6%)
Asian American	1 (1.8%)
**Localized or metastatic (at time PD1i initiated)**	
Advanced, localized disease	5 (8.9%)
Metastatic disease	51 (91.1%)
**Tumor pathology**	
Liposarcoma	11 (19.6%)
Leiomyosarcoma	7 (12.5%)
Synovial sarcoma	4 (7.1%)
Chordoma	4 (7.1%)
Spindle cell sarcoma	4 (7.1%)
Osteosarcoma	3 (5.4%)
Undifferentiated pleomorphic sarcoma	3 (5.4%)
Other^a^	20 (35.7%)
**Primary site of tumor**	
Intra-abdominal	15 (26.8%)
Lower extremity	13 (23.2%)
Trunk	11 (19.6%)
Intra-thoracic	4 (7.1%)
Head/neck	4 (7.1%)
Uterus	3 (5.4%)
Upper extremity	2 (3.6%)
Other/unknown	4 (7.1%)
**Immunotherapy drug(s) received**	
Nivolumab alone	30 (53.6%)
Pembrolizumab alone	20 (35.7%)
Nivolumab and ipilimumab combination^b^	6 (10.7%)
**Number of immunotherapy cycles received**	
Four or less cycles	33 (58.9%)
Greater than four cycles	23 (41.1%)
**Other agent given with immunotherapy**	
Immunotherapy alone	42 (75%)
Immunotherapy with secondary agent	14 (25%)
**# of prior therapies**	
Two or less	29 (51.8%)
More than two	27 (48.2%)
**PD1 status**	
Positive	17 (30.4%)
Negative	8 (14.3%)
Unknown	31 (55.4%)
**PDL1 status**	
Positive	8 (14.3%)
Negative	18 (32.1%)
Unknown	30 (53.6%)

^a^The “Other” tumor pathology group includes the following sarcoma subtypes: alveolar soft part (*n* = 1), angiosarcoma (*n* = 1), chondrosarcoma (*n* = 1), Ewing (*n* = 1), epitheliod angiosarcoma (*n* = 1), epitheliod sarcoma (*n* = 2), gastrointestinal stromal tumor (*n* = 2), inflammatory myofibroblastic tumor (n = 1), interdigitating dendritic cell (*n* = 1), mesenchymal chondrosarcoma (*n* = 1), myoepithlioma (*n* = 1), myxoinflammatory fibroblastic soft tissue (*n* = 1), myxofibrosarcoma (*n* = 1), myxoid sarcoma (*n* = 1), sarcomatoid carcinoma (*n* = 2), solitary fibrous (*n* = 1), and undefined soft tissue (*n* = 1)

^b^Three of these patients were treated with nivolumab and ipilimumab the entire duration of studied time, two of the patients started with nivolumab alone followed by nivolumab and ipilimumab combination, and one patient started with nivolumab and ipilimumab followed by nivolumab alone

The majority of patients had metastatic disease at the time PD1i therapy was initiated (91.1%), however, five had localized disease only (8.9%). The histologies of these patients sarcomas were diverse; the largest single subset was LPS (*n* = 11), followed by leiomyosarcoma (LMS) (*n* = 7), synovial sarcoma (*n* = 4), chordoma (*n* = 4), spindle cell sarcoma (*n* = 4), osteosarcoma (*n* = 3), UPS (*n* = 3), and other (*n* = 20). The most common primary site of these tumors was intra-abdominal (*n* = 15), followed by the lower extremity (*n* = 13), trunk (*n* = 11), intra-thoracic (*n* = 4), head/neck (*n* = 4), uterus (*n* = 3), upper extremity (*n* = 2), and other (*n* = 4). The majority of patients who received immunotherapy had already been treated with chemotherapy and had a median of two prior regimens (range of 0–8). Thirty patients received nivolumab and 20 received pembrolizumab. Six patients received a combination of nivolumab and ipilimumab (a CTLA-4 targeted checkpoint inhibitor), with two of these patients initially starting on nivolumab alone before switching to this dual checkpoint inhibitor treatment; one of these six patients transitioned to nivolumab treatment alone. Patients received a median of three cycles of PD1i treatment, most commonly discontinued due to progression of disease and/or death.

Of the 25 tumors that had PD1 status reported, 17 tumors were positive, and 8 tumors were negative. Moreover, 26 tumors had PDL1 status reported, of which 8 were positive, and 18 were negative.

### Partial response to PD1 inhibitors seen in a subset of sarcoma patients

**Fig. 1:**
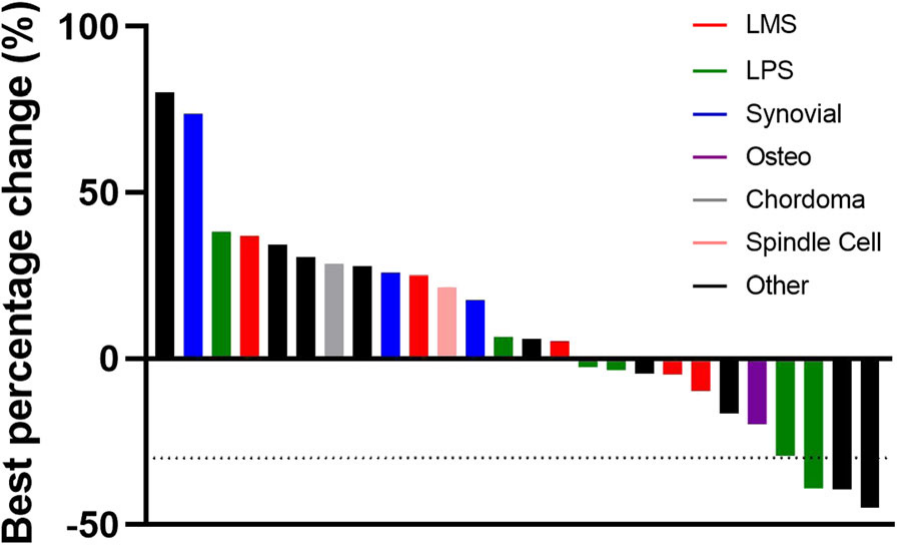
Waterfall plot of best tumor response to PD1i. Histogram bars each represent the best percent decrease in tumor burden seen in that individual patient, or if no decrease was observed, the closest target tumor burden to baseline measurement when PD1i was started. Bar colors correlate with the individual tumor type. The dotted line indicates a 30% decrease in tumor burden; bars reaching below this are indicative of patients having partial response to PD1i

Based on imaging/monitoring criteria, 26 patients were eligible for PFS analysis and measurement of the best tumor burden response. No complete responses were observed. Partial responses (at least 30% regression in target tumor burden) were seen in three patients; one patient with LPS who was treated with single-agent nivolumab (unknown PD1 and PDL1 status), one with inflammatory myofibroblastic sarcoma who was treated with single-agent nivolumab (positive PD1, but negative PDL1 status), and one with sarcomatoid carcinoma who was treated with single-agent pembrolizumab (positive PD1 and PDL1 status) (Fig. 1). The overall response rate of these 26 patients was 11.5%.

### PFS trends in sarcoma patient immunotherapy recipients

**Fig. 2:**
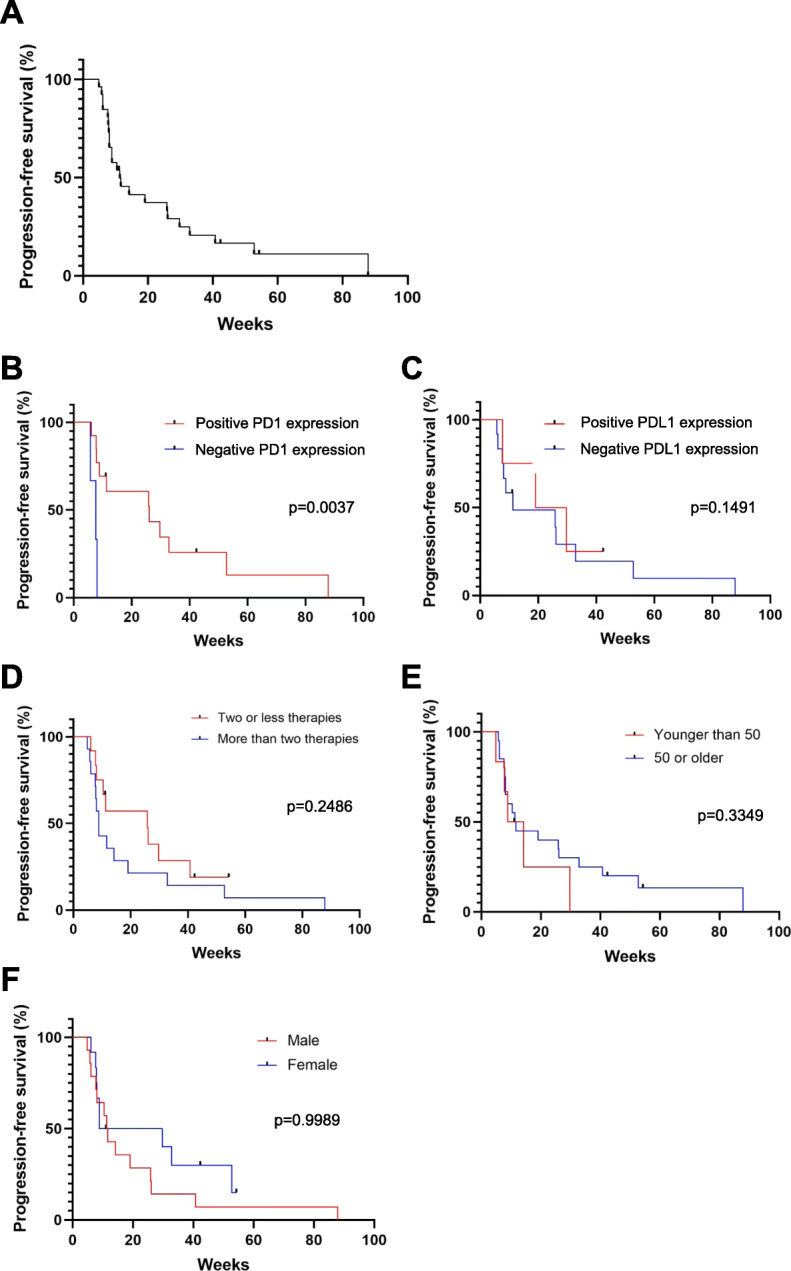
Rates of progression-free survival. Kaplan-Meier graphs are shown demonstrating the progression-free survival rates of each of the denoted groups while on PD1i. **a** All subjects analyzed together are shown. Groups are separated into (**b**) presence of PD1 expression, (**c**) presence of PDL1 expression, (**d**) number of prior systemic therapies, (**e**) age, and (**f**) sex. Each hash mark denotes when a single patient was censored from analysis. Patient groups were compared by log-rank (Mantel-Cox) analysis

The median PFS of all analyzed patients was 11.3 weeks and the 90 day PFS rate was 48.8% (Fig. 2a). Separating the patients into groups according to which immunotherapy they received, there was no significant difference in median PFS seen between these groups (*p* = 0.4831) (Additional file 1a). Of the patients who had PD1 tumor expression testing, positive expression of PD1 was significantly associated with improved PFS versus those whose tumors were negative for PD1 expression (26 versus 7.6 weeks; *p* = 0.0037) (Fig. 2b). On the other hand, patients whose tumors expressed PDL1 did not appear to have a statistically significant difference in PFS versus non-expressers (24.4 versus 11.3 weeks; *p* = 0.1491) (Fig. 2c). Of note, patients who were pretreated with more than two other anti-neoplastic systemic therapies had no significant difference in PFS compared to those who received two or less prior therapies (8.9 versus 25.9 weeks; *p* = 0.2486) (Fig. 2d). Patients who were younger than 50 years were not found to have different rates of PFS compared to those 50 years and older (11.5 versus 11.4 weeks; *p* = 0.3349) (Fig. 2e). Additionally, there was no difference in PFS when adjusted by sex (11.4 versus 19.3 weeks; *p* = 0.9989) (Fig. 2f).

### OS trends in sarcoma patient immunotherapy recipients

**Fig. 3:**
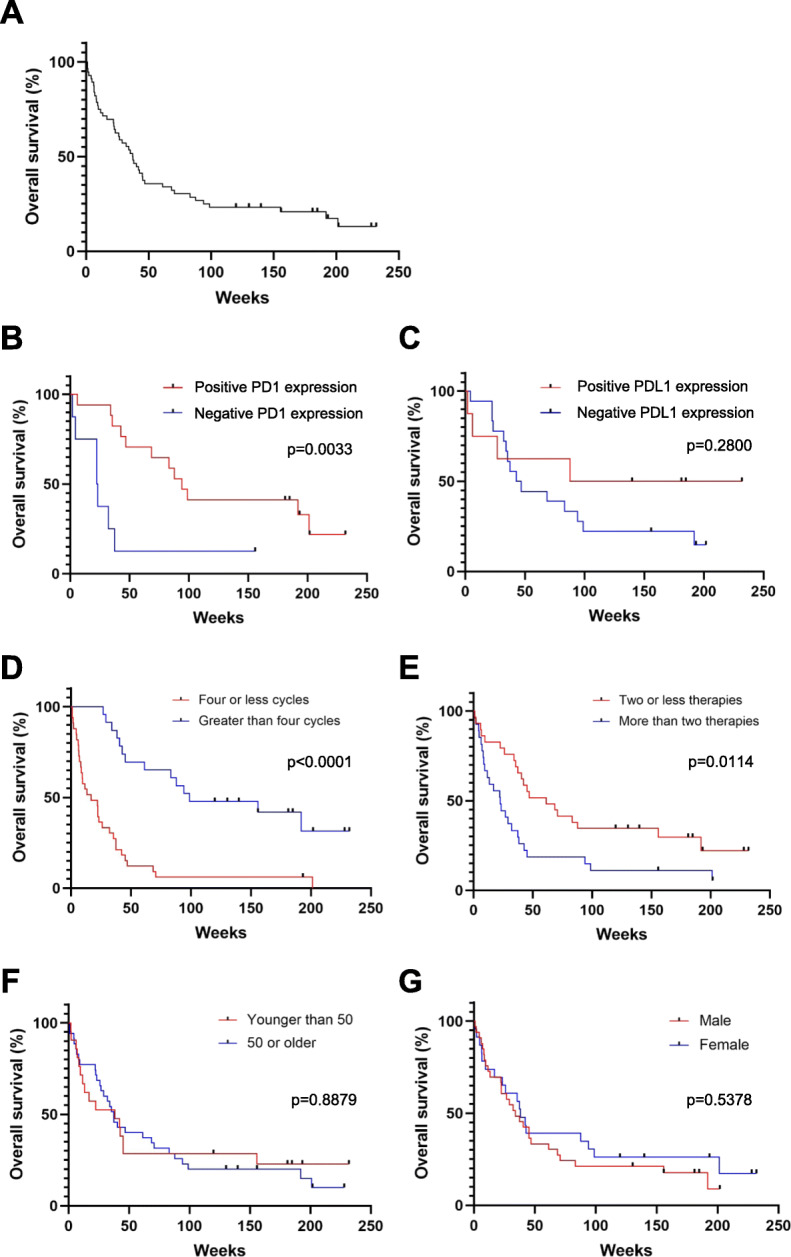
Rates of overall survival. Kaplan-Meier graphs are shown demonstrating the overall survival rates of each of the denoted groups while on PD1i. **a** All subjects analyzed together are shown. Groups are separated into (**b**) presence of PD1 expression, (**c**) presence of PDL1 expression, (**d**) number of PD1i cycles, (**e**) number of prior systemic therapies, (**f**) age, and (**g**) sex. Each hash mark denotes when a single patient was censored from analysis. Patient groups were compared by log-rank (Mantel-Cox) analysis

The median OS of all PD1i-treated patients was 37.4 weeks from the day that PD1i therapy was initiated (Fig. 3a). There was no significant difference in median OS when the patients were separated into groups according to which treatment they received (*p* = 0.4004) (Additional file 1b). In comparing these patients, it was found that those who tested positive for PD1 tumor expression had a significantly higher median OS than those who tested negative (94.14 versus 22.9 weeks; *p* = 0.0033) (Fig. 3b). Alternatively, there was no statistical OS difference between patients who had PDL1 positive tumors compared to those who had PDL1 negative tumors (159.7 versus 44.8 weeks; *p* = 0.2800) (Fig. 3c). Patients who were treated with four or less cycles of PD1i had a significantly lower median OS than those who received more than 4 cycles (16.7 versus 98.9 weeks; *p* < 0.0001) (Fig. 3d). Those patients who received more than two prior anti-neoplastic systemic therapies had a lower median OS than those receiving fewer than two therapies prior to initiating PD1i therapy (61.1 versus 22.4 weeks; *p* = 0.0114) (Fig. 3e). There were no significant differences between patients who were younger than 50 years compared to those that were 50 years or older, median OS (38.1 versus 37.3 weeks; *p* = 0.8879) (Fig. 3f). Additionally, male or female gender was not associated with a difference in OS (34.1 versus 38.1 weeks; *p* = 0.5378) (Fig. 3g).

### Adverse events in patients receiving PD1i therapy

In accord with previous studies of PD1i treatment, we found that adverse events using immune checkpoint inhibitors are moderately common [37, 38], but were typically grade 1–2 with fatigue (*n* = 31) being the most common, followed by hypothyroidism (*n* = 2), colitis (*n* = 2), transaminitis (*n* = 2), endocarditis (*n* = 1), pleural effusion (*n* = 1), pneumonitis (*n* = 1), and uveitis (*n* = 1) (Table 2). Grade 3 level adverse events observed in this cohort included fatigue (*n* = 5), colitis (*n* = 1), transaminitis (*n* = 1), hypocalcemia (*n* = 1), hyponatremia (*n* = 1), pancreatitis (*n* = 1), rash (*n* = 1), and pneumonitis (*n* = 1), all of which have been documented in previous studies with PD1i therapy [37, 38]. There were also three incidents of ungraded adverse events, including fatigue (*n* = 1), enteritis (*n* = 1), and psoriasis (*n* = 1).

**Table 2:** Adverse events

	Grade 1–2	Grade 3	Grade 4	Grade 5	Ungraded
Fatigue	31 (55.4%)	5 (8.9%)	–	–	1 (1.8%)
Colitis	2 (3.6%)	1 (1.8%)	–	–	–
Transaminitis	2 (3.6%)	1 (1.8%)	–	–	–
Hypothyroidism	2 (3.6%)	–	–	–	–
AIHA	–	–	–	1 (1.8%)	–
Hypocalcemia	–	1 (1.8%)	–	–	–
Hyponatremia	–	1 (1.8%)	–	–	–
Pancreatitis	–	1 (1.8%)	–	–	–
Rash	–	1 (1.8%)	–	–	–
Endocarditis	1 (1.8%)	–	–	–	–
Pleural effusion	1 (1.8%)	–	–	–	–
Pneumonitis	1 (1.8%)	1 (1.8%)	–	–	–
Uveitis	1 (1.8%)	–	–	–	–
Enteritis	–	–	–	–	1 (1.8%)
Psoriasis	–	–	–	–	1 (1.8%)

Most importantly, there was a grade 5 adverse event (death), which was reported as likely attributable to autoimmune hemolytic anemia (AIHA) secondary to initial treatment with nivolumab and ipilimumab. This patient was a 32-year-old male with a history of epithelioid sarcoma of the right gluteus who was previously treated with surgical resection, chemotherapy, and radiation before initiation of PD1i therapy. He received one dose of ipilimumab and nivolumab and presented the following day with AIHA; this anemia was found to be refractory to treatment with high-dose steroids and intravenous immunoglobulin. Plasmapheresis treatment was planned; however, the patient suffered disseminated intravascular coagulation and cardiac arrest prior to this which led to his death.

## Discussion

There remains a strong and unmet need regarding treatment options available for advanced sarcoma patients. Recently, checkpoint inhibitors have become an attractive option in a variety of cancers. Prior studies of checkpoint inhibitors in advanced sarcomas have shown promising results, especially in some sarcoma subtypes, such as UPS and LPS [35, 36, 39–41]. The interest in utilizing checkpoint inhibitors remains strong, with several ongoing phase II PD1i trials, including the SARC 032 trial [39], the NEXIS trial (NCTT03116529), and the SAINT trial (NCT03138161).

In this study, we retrospectively analyzed the outcomes of sarcoma patients who received PD1i therapy and correlated their demographic and disease-specific information to their PFS and OS rates. Looking at the patients eligible for PFS evaluation, three unique patients were found to have partial responses to PD1i. The patient with the most robust recorded response (44.8% tumor regression) was a 21-year-old woman with metastatic inflammatory myofibroblastic sarcoma (with ALK fusion) who received three cycles of nivolumab plus crizotinib before progression. Interestingly, our patient’s tumor was PDL1 negative and PD1 positive. While no prospective studies have described increased PD1 positivity in these tumors, nor has the therapeutic use of immune checkpoint inhibitors have been reported, multiple case reports have found inflammatory myofibroblastic sarcomas to express PDL1 at a significant level and have notable CD8+ tumor-infiltrating lymphocyte populations [42, 43].

The patient with the next best response (39.2% tumor regression) was a 56-year-old woman with metastatic carcinosarcoma of the alveolar ridge of the jaw who received 13 cycles of pembrolizumab plus pazopanib prior to progression. This patient’s tumor was positive for both PD1 and PDL1 expression, which follows suit with prior studies [44]. It should also be noted that there are several case reports of checkpoint inhibitors being successfully used in lung sarcomatoid carcinoma treatment [45, 46].

The last patient to show a substantial response (39.1% tumor regression) to PD1i was a 62 year old woman with localized de-differentiated LPS who received at least 12 cycles of nivolumab (patient was lost to follow-up/screened after she moved re-establishing with new oncologist). The lymphocytic make-up of these tumors has been explored and due to their immunogenic nature, it has been strongly suggested that checkpoint inhibitors may be a treatment option of interest [47]. The SARC028 trial enrolled multiple patients with de-differentiated LPS and found that this subset had about a 10% overall response rate and 44% 12-week PFS rate. Therefore, it appears that some de-differentiated LPS have the potential to respond to PD1i, but it is unclear how to differentiate which will be responders versus non-responders [35]. Thus, the development of better biomarkers to predict the response of sarcomas to checkpoint inhibitors are needed.

Of the patients studied, there was a median PFS rate of 11.3 weeks, with 48.8% having a 90 day PFS rate. Using the previously recommended three-month PFS rate of ≥40% being suggestive of modest drug activity for sarcoma second-line therapy [48], our findings suggest that checkpoint inhibitor treatment would meet these drug activity criteria. These findings are also in line with the SARC028 study results showing their patients to have a 3 month PFS rate of 55% [35]. Alternatively, Alliance A091401 studying checkpoint inhibitors use in metastatic sarcoma patients did not report a PFS rate ≥ 40% at 3 months in their nivolumab alone treatment group but did show a PFS rate of 53.7% in patients treated with nivolumab and ipilimumab [36].

The median OS in our study was 37.4 weeks, which contrasts with the median overall survival of 49 weeks reported in the SARC028 trial [39] and the results of the Alliance A091401 trial that showed a median overall survival of 46.4 weeks in the nivolumab arm versus 62.1 weeks in the nivolumab plus ipilimumab arm [36]. The differences between our reported data and these other prospective studies was mainly due to the sarcoma subtypes included and the reality that our patients were heavily pretreated. Our findings suggest a significantly longer OS rate in patients who had not received more than two prior anti-neoplastic therapies prior to PD1i treatment; and OS rates in prospective studies where patients were not as heavily pre-treated may be higher. It should also be noted that this finding does not necessarily suggest that PD1i therapies are inherently more effective in patients with fewer prior treatment regimens, as sarcoma patients early in their treatment history logically would have a greater amount of time until death than someone on a much later line of therapy. Future studies would benefit from greater numbers of patients, which would allow them to be stratified into distinct groups, including those with different levels of pre-treatment. Of the PD1i-treated patients who had PD1 testing, positive expression of PD1 was found to be positively associated with longer PFS and OS than those who did not have PD1 tumor expression. In looking at the few patients with a verified negative PD1 test in this study, none had a partial response or even stable disease with PD1i treatment. This suggests that intratumoral PD1 status may have a very important role in determining which patients will have substantial response to PD1i. Expression of PD1 is not seen on tumor cells themselves, but instead primarily on the immune cell infiltrate within and surrounding the tumor. It is unknown from the available testing if the PD1 positive tumors were positive largely due to effector CD4+ or CD8+ T cell expression, or perhaps even due to other PD1-expressing immune cells, such as NKT cells, activated monocytes, or B cells [49]. What can be suggested from these findings is that tumors testing positive for PD1 expression have an identifiable immune cell population and that this may cause them to be more immunologically active tumors, as opposed to the immunologically deserted environments of other tumors. Clinical trials have begun to address these questions by analyzing tumor biopsy specimens, such as those from the SARC028 trial which suggested that higher pre-treatment presence of effector CD8+ T cells and T regulatory cells can positively correlate with response to PD1i treatment [50].

This same association was not seen with PDL1, possibly because patients were treated with a PD1 as opposed to a PDL1-targeted therapy. As the most common reason for PD1i discontinuation was progression of disease (only four patients needed to be discontinued from therapy due to adverse events secondary to treatment), it was somewhat expected that patients treated with a fewer number of PD1i cycles had lower rates of PFS and OS. Sex and age differences were also examined; however, no relationship between these variables and PFS or OS was apparent. It should be noted that 14 (25%) patients received secondary agents in addition to PD1i in this study. This group of patients was directly compared to the PD1i alone group and no significant differences in PFS and OS were found (data not shown). Additionally, removal of these patients from the data sets shown in Figs. 2 and 3 did not change the statistically significant differences that were found between groups (data not shown).

Overall, the adverse events noted in this study were consistent with the types, frequencies, and grades of adverse events described in other clinical PD1 studies [38, 51]. For example, the majority of adverse events were grade 1 or 2, and the most frequent adverse event was fatigue, which was reported in over half of the patients. All of the adverse events reported were ones previously described in other PD1 studies; alternatively, the most historically uncommon event to have been observed in our study was a grade 5 incident of AIHA. In a recent report, there have been at least 68 cases of AIHA described to be secondary to treatment with immune checkpoint inhibitors [52]. Of the two reported AIHA cases that were fatal, both patients were men and involved treatment with nivolumab; these attributes were shared with our case as well. While rare, this unfortunate case highlights the importance of early detection and treatment of AIHA in the setting of PD1i therapy.

Soft-tissue sarcomas represent a diverse group of tumors with a wide range of survival times, such as seen with this study’s patient group (time from diagnosis to death ranging from 5 months to 28 years). Due to the small number of patients studied on PD1i therapy thus far and our ever-growing understanding of checkpoint inhibitor pharmacology, it is difficult to determine which sarcoma subtypes may benefit optimally from therapy and which are most likely to have significant adverse events. To date, checkpoint inhibitor responses have been documented in many sarcoma pathologies, including clear cell sarcoma, synovial sarcoma, osteosarcoma, chondrosarcoma, gastrointestinal stromal tumor (GIST), UPS, LMS, angiosarcoma, and myxofibrosarcoma [35, 39, 53]. It is also possible that other tumor variables may be affecting PD1i responsiveness; for example, the presence of microsatellite instability is strongly correlated to the checkpoint inhibitor responses in other malignancies [54, 55]. Prior studies suggest that microsatellite instability may play a role in sarcoma pathogenesis [56, 57] and may also affect checkpoint inhibitor response [58].

This study supports prior research that PD1i therapy has an important role in the treatment of sarcomas; however, it is also essential to evaluate what may render PD1i even more efficacious. For example, breast, non-small cell lung, and other cancers have National Comprehensive Cancer Network-approved indications for checkpoint inhibitor use in combination with chemotherapy [59, 60]. There are currently several ongoing sarcoma clinical trials to study the addition of checkpoint inhibitors with other systemic therapies, such as cyclophosphamide (NCT02406781), nab-rapamycin (NCT03190174), and axitinib [61]. The later combination was recently published, revealing promising results particularly for patients with alveolar soft-part sarcomas [61]. It is also possible that immune checkpoint blockade may be more efficacious in the setting of chemotherapy induction, which has been helpful in other less intrinsically immunogenic cancer variations [62]. The combination of checkpoint inhibitors with T-cell immunotherapies has also been proposed, which may induce further beneficial responses [63, 64].

### Limitations

This study had several limitations. First, this was a single-institution, retrospective analysis with a relatively small sample size. Second, some patients were treated with a variety of PD1 therapies and/or other agents (e.g. monoclonal antibodies) too varied to account for in our analyses. Additionally, this study is limited in that over 50% of the patients who received immunotherapy did not have tumors that were tested for PD1 or PDL1 status; therefore, our results are only representative of a small group of sarcomas. The actual percentage of sarcomas expressing PD1 and/or PDL1 is also unknown, with studies suggestion that 4–12% of sarcomas have positive expression of these markers [35, 65]. It is possible that PD1 and/or PDL1 expression is also more common in certain types of sarcomas, such as UPS and LPS [35, 66]. These caveats notwithstanding, in our cohort, PD1i-treated patients who had positive expression of PD1 or PDL1 had longer PFS and OS than those who did not have PD1 or PDL1 expression. These findings are an important addition to the literature and should be further investigated in larger populations of sarcoma patients.

## Conclusions

This retrospective study suggests activity of PD1i in a pretreated cohort of advanced sarcoma patients, particularly in those with PD1 positive tumors. Our results highlight the importance of further research to better identify the optimal target population and biomarkers of response.

## Supplementary information

**Additional file 1.** Rates of survival analyzed by treatment regimen. Kaplan-Meier graphs are shown demonstrating the progression-free (A) and overall (B) survival rates of each of the denoted groups while on PD1i. Each hash mark denotes when a single patient was censored from analysis. Patient groups were compared by log-rank (Mantel-Cox) analysis.

## Data Availability

The datasets used and/or analyzed during the current study are available from the corresponding author upon a reasonable request.

## Abbreviations

AIHA: Autoimmune hemolytic anemia
CT: Computed tomography
CTLA-4: Cytotoxic T-lymphocyte-associated protein 4
GIST: Gastrointestinal stromal tumor
LMS: Leiomyosarcoma
LPS: Liposarcoma
MRI: Magnetic resonance imaging
OS: Overall survival
PD1: Programmed cell death protein 1
PD1i: Programmed cell death protein 1 inhibitor
PDL1: Programmed death-ligand 1
PFS: Progression-free survival
RECIST: Response Evaluation Criteria in Solid Tumors
UPS: Undifferentiated pleomorphic sarcoma
