# Supplementary figures and images for: Activity of PD1 inhibitor therapy in advanced sarcoma: a single-center retrospective analysis
Source: BMC Cancer. 2020 Jun 5;20:527. doi: 10.1186/s12885-020-07021-x (PMC7275332; doi:10.1186/s12885-020-07021-x)

A

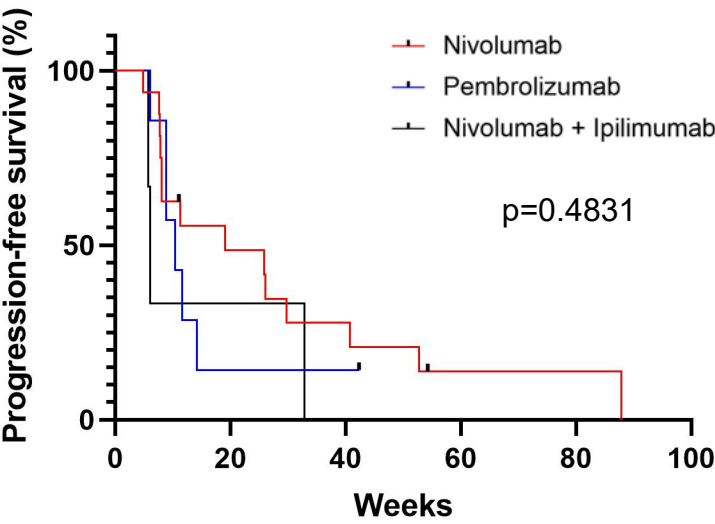

B

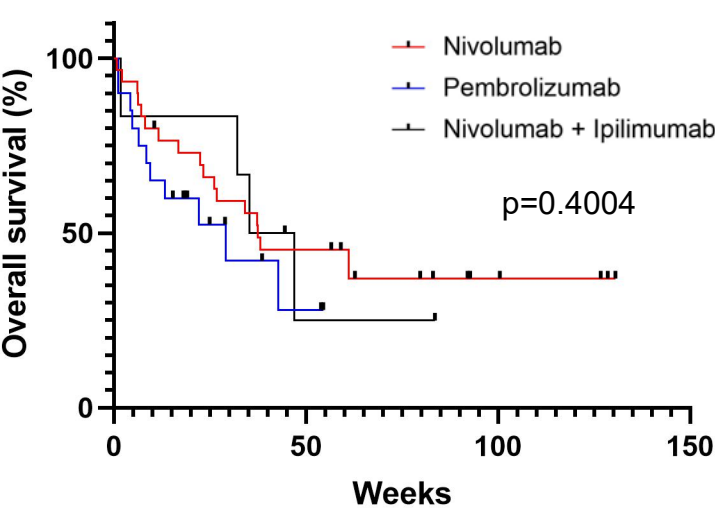

Supplement: Supplementary file 1 — Additional file 1. Rates of survival analyzed by treatment regimen. Kaplan-Meier graphs are shown demonstrating the progression-free (A) and overall (B) survival rates of each of the denoted groups while on PD1i. Each hash mark denotes when a single patient was censored from analysis. Patient groups were compared by log-rank (Mantel-Cox) analysis. [file 12885_2020_7021_MOESM1_ESM.pdf]
